# Performance of a [^18^F]Flortaucipir PET Visual Read Method Across the Alzheimer Disease Continuum and in Dementia With Lewy Bodies

**DOI:** 10.1212/WNL.0000000000207794

**Published:** 2023-11-07

**Authors:** Emma M. Coomans, Lotte A. de Koning, Roos M. Rikken, Sander C.J. Verfaillie, Denise Visser, Anouk den Braber, Jori Tomassen, Marleen van de Beek, Lyduine E. Collij, Afina W. Lemstra, Albert D. Windhorst, Frederik Barkhof, Sandeep S.V. Golla, Pieter Jelle Visser, Philip Scheltens, Wiesje M. van der Flier, Rik Ossenkoppele, Bart N.M. van Berckel, Elsmarieke van de Giessen

**Affiliations:** From the Radiology & Nuclear Medicine (E.M.C., L.A.d.K., R.M.R., S.C.J.V., D.V., L.E.C., A.D.W., F.B., S.S.V.G., B.N.M.v.B., E.v.d.G.), Vrije Universiteit Amsterdam, Amsterdam UMC location VUmc; Brain Imaging (E.M.C., L.A.d.K., R.M.R., S.C.J.V., D.V., L.E.C., A.D.W., F.B., S.S.V.G., B.N.M.v.B., E.v.d.G.), Amsterdam Neuroscience; Medical Psychology (S.C.J.V.), Amsterdam UMC location University of Amsterdam; Alzheimer Center Amsterdam (A.d.B., J.T., M.v.d.B., A.W.L., P.J.V., P.S., W.M.v.d.F., R.O.), Neurology, Vrije Universiteit Amsterdam, Amsterdam UMC location VUmc; Neurodegeneration (A.d.B., J.T., M.v.d.B., A.W.L., P.J.V., P.S., W.M.v.d.F., R.O.), Amsterdam Neuroscience; Department of Biological Psychology (A.d.B.), Vrije Universiteit Amsterdam, the Netherlands; Queen Square Institute of Neurology and Centre for Medical Image Computing (F.B.), University College London, United Kingdom; Alzheimer Center Limburg (P.J.V.), School for Mental Health and Neuroscience, Maastricht University, the Netherlands; Division of Neurogeriatrics (P.J.V.), Department of Neurobiology, Care Sciences and Society, Karolinska Institutet, Stockholm, Sweden; Department of Epidemiology & Data Science (W.M.v.d.F.), Vrije Universiteit Amsterdam, Amsterdam UMC, the Netherlands; and Clinical Memory Research Unit (R.O.), Lund University, Sweden.

## Abstract

**Background and Objectives:**

Recently, the US Food and Drug Administration approved the tau-binding radiotracer [^18^F]flortaucipir and an accompanying visual read method to support the diagnostic process in cognitively impaired patients assessed for Alzheimer disease (AD). Studies evaluating this visual read method are limited. In this study, we evaluated the performance of the visual read method in participants along the AD continuum and dementia with Lewy bodies (DLB) by determining its reliability, accordance with semiquantitative analyses, and associations with clinically relevant variables.

**Methods:**

We included participants who underwent tau-PET at Amsterdam University Medical Center. A subset underwent follow-up tau-PET. Two trained nuclear medicine physicians visually assessed all scans. Inter-reader agreement was calculated using Cohen κ. To examine the concordance of visual read tau positivity with semiquantification, we defined standardized uptake value ratio (SUVr) positivity using different threshold approaches. To evaluate the prognostic value of tau-PET visual read, we performed linear mixed models with longitudinal Mini-Mental State Examination (MMSE).

**Results:**

We included 263 participants (mean age 68.5 years, 45.6% female), including 147 cognitively unimpaired (CU) participants, 97 amyloid-positive participants with mild cognitive impairment or AD dementia (AD), and 19 participants with DLB. The visual read inter-reader agreement was excellent (κ = 0.95, CI 0.91–0.99). None of the amyloid-negative CU participants (0/92 [0%]) and 1 amyloid-negative participant with DLB (1/12 [8.3%]) were tau-positive. Among amyloid-positive participants, 13 CU participants (13/52 [25.0%]), 85 with AD (85/97 [87.6%]), and 3 with DLB (3/7 [42.9%]) were tau-positive. Two-year follow-up visual read status was identical to baseline. Tau-PET visual read corresponded strongly to SUVr status, with up to 90.4% concordance. Visual read tau positivity was associated with a decline on the MMSE in CU participants (β = −0.52, CI −0.74 to −0.30, *p* < 0.001) and participants with AD (β = −0.30, CI −0.58 to −0.02, *p* = 0.04).

**Discussion:**

The excellent inter-reader agreement, strong correspondence with SUVr, and longitudinal stability indicate that the visual read method is reliable and robust, supporting clinical application. Furthermore, visual read tau positivity was associated with prospective cognitive decline, highlighting its additional prognostic potential. Future studies in unselected cohorts are needed for a better generalizability to the clinical population.

**Classification of Evidence:**

This study provides Class II evidence that [^18^F]flortaucipir visual read accurately distinguishes patients with low tau-tracer binding from those with high tau-tracer binding and is associated with amyloid positivity and cognitive decline.

## Introduction

Alzheimer disease (AD) is pathologically characterized by β-amyloid (Aβ) plaques and neurofibrillary tau tangles.^1^ The clinical application of biomarkers for Aβ pathology have substantially improved the diagnostic process of AD dementia, leading to increased diagnostic confidence and changes in treatment strategies.^2,3^ However, PET biomarkers for tau pathology have shown higher specificity for AD dementia^4-6^ because the presence of incidental or comorbid Aβ pathology is common, especially at older age and in *APOE* ε4 carriers.^7,8^ Moreover, tau-PET is more strongly associated with cognitive decline and atrophy.^9,10^ Therefore, tau-PET holds potential to become an important diagnostic and prognostic tool in the clinic.

Recently, the US Food and Drug Administration (FDA) approved the tau-binding radiotracer [^18^F]flortaucipir and an accompanying visual read method to support the diagnostic process in cognitively impaired patients assessed for AD. The US FDA–approved visual read method defines increased tracer binding in late-stage tau regions (corresponding to tau-PET Braak stages IV–VI^11,12^) as tau-positive, whereas increased tracer binding isolated to early-stage tau regions (corresponding to tau-PET Braak stages I–III^11,12^) or absence of increased tracer binding is defined as tau-negative. As a result, this method has strong specificity for AD, given the focus on late-stage tau regions.^13^ Moreover, this method provides valuable prognostic information.^14^ However, studies evaluating the performance of this visual read method in independent samples are limited.

The aim of this study was to evaluate the performance of the US FDA–approved [^18^F]flortaucipir PET visual read method, by determining its reliability, accordance with semiquantitative analyses, and associations with clinically relevant variables. We included participants along the AD continuum and participants with dementia with Lewy bodies (DLB) because AD-type tau tangles are observed in approximately 50% of patients with DLB.^15^ To evaluate key properties of the method, we assessed inter-reader agreement, examined longitudinal stability of the method, and compared it with a semiquantitative measure of tracer binding. To evaluate the method in relation to clinically relevant variables, we examined associations with clinical diagnosis, Aβ status, demographic factors, and prospective cognitive decline. The primary research questions addressed in this study are as follows: is the visual read method reliable, and is tau-PET visual read associated with clinical diagnosis, Aβ status, and cognitive decline.

## Methods

### Participants

We included 263 participants who underwent [^18^F]flortaucipir PET between 2015 and 2021 for research purposes at Amsterdam University Medical Center (Amsterdam, the Netherlands). The study population largely consisted of participants from the Amsterdam Dementia Cohort,^16,17^ the Subjective Cognitive Impairment Cohort,^18^ the Dementia with Lewy Bodies Project,^19^ and the Amsterdam substudy of the EMIF-AD PreclinAD study.^20^ From these cohorts, cognitively normal identical twins (n = 82) and cognitively normal participants with subjective cognitive decline (SCD; n = 56) were included along with cognitively impaired participants with mild cognitive impairment (MCI; n = 12), those with probable AD dementia (n = 85), and those with DLB (n = 19). All participants with MCI and probable AD dementia had positive Aβ PET and/or CSF biomarkers.^21,22^ Furthermore, the study population consisted of 9 healthy controls who were not part of the aforementioned cohorts but who underwent tau-PET as control participants in prior PET kinetic modeling studies.^23,24^ Details are described in the eMethods (links.lww.com/WNL/D110). Exclusion criteria for undergoing tau-PET included large structural abnormalities on MRI, a history of severe traumatic brain injury, and (prior) use of Aβ-lowering or tau-lowering drugs.

Participants were categorized into 3 groups based on their clinical presentation: (1) cognitively unimpaired (CU) participants (those with SCD, twins, and healthy controls), (2) cognitively impaired participants with AD (Aβ-positive participants with MCI and those with probable AD dementia, hereafter referred to as “AD”), and (3) participants with DLB. All participants had cross-sectional Mini-Mental State Examination (MMSE; global cognitive functioning) available, and 140 participants had prospective 1.5 ± 1.7 years follow-up MMSE available. A total of 594 MMSE scores (number of visits per participant 1–7 [median 2], time between visits varied per participant) were included.

### Standard Protocol Approvals, Registrations, and Patient Consents

All participants provided written informed consent. All studies were approved by the Medical Ethics Review Committee of the VU University Medical Center (Amsterdam, the Netherlands).

### Aβ Status

Aβ status of CU participants was determined by [^18^F]florbetapir or [^18^F]flutemetamol PET visual read according to company guidelines. Aβ status of participants with AD and DLB were determined at diagnostic screening by either PET visual read ([^18^F]florbetapir, [^18^F]flutemetamol, and [^18^F]florbetaben according to company guidelines or [^11^C]PiB according to previously published methods^25^) or CSF using previously determined cutoffs.^26^ If both PET and CSF were available, PET was chosen. We used Aβ status that was available in closest proximity in time to tau-PET. Aβ status was missing for 3 CU participants.

### Tau-PET and MRI Acquisition

All participants underwent baseline tau-PET. A subset (n = 50 CU participants and n = 40 with AD) underwent 2.1 ± 0.5 years of follow-up tau-PET of which 15 CU participants additionally underwent 4.5 ± 0.4 years of follow-up tau-PET. All scans were acquired using a dual time point dynamic protocol, starting immediately after [^18^F]flortaucipir administration and including at least the 0–30 minutes and 80–100 minutes postinjection time interval.^27,28^ All scans were acquired on a Philips TF-64 PET/CT scanner (baseline: n = 244 Philips Ingenuity and n = 19 Philips Gemini; follow-up: n = 105 Philips Ingenuity; Philips Medical System, Best, the Netherlands). Low-dose CT scans were acquired before both parts of the dynamic scan for attenuation correction purposes. Participants underwent 3-dimensional T1-weighted MRI on a 3T scanner for coregistration and brain region-of-interest purposes.

### Tau-PET Visual Read

Tau-PET scans were prepared and visually read according to US FDA–approved guidelines.^13^ First, dynamic PET frames were summed from 80 to 100 minutes postinjection. T1-weighted MRIs were then coregistered to the corresponding summed image using Vinci software (Max Planck Institute, Cologne, Germany). Scans were reoriented to remove head tilt. Background activity was determined by calculating the mean counts in the cerebellum (manually delineated in the transversal plane at the maximum cross-sectional area). Voxels of increased activity were defined as >65% above the cerebellar average. Following US FDA–approved guidelines, increased activity in posterolateral temporal, occipital, or parietal/precuneus region(s) in either hemisphere, with or without frontal involvement, resulted in a positive visual read. The absence of increased activity or increased activity isolated to medial temporal, anterolateral temporal, and/or frontal regions resulted in a negative visual read. Patterns of isolated or small nonconfluent foci of increased activity were not defined as tau-positive.

All scans were visually read by 2 trained nuclear medicine physicians (B.v.B. and E.v.d.G.) blinded to clinical information. The 2 readers gave confidence scores for each scan ranging from 1 (lowest confidence) to 5 (highest confidence). Scans were presented in a random order. The 2 nuclear medicine physicians were first trained with a test set of 20 randomly selected baseline scans. The 20 test set scans were visually read for a second time within the complete set of 263 baseline scans, from which intra-reader agreement was determined. Subsequently, the 105 follow-up scans were assessed. Scans with between-reader disagreement were re-read by the 2 nuclear medicine physicians in a joint consensus meeting resulting in a consensus read.

### Tau-PET Standardized Uptake Value Ratio

To compare tau-PET visual read with a semiquantitative measure of tracer binding, we calculated standardized uptake value ratios (SUVrs) using whole cerebellar gray matter as reference region in 2 regions-of-interests (ROIs) based on the Hammers and Svarer atlasses.^29,30^ First, we calculated SUVr in a temporal meta-ROI corresponding to a volume-weighted average of the bilateral entorhinal cortex, amygdala, parahippocampal gyrus, fusiform gyrus, and middle, inferior and superior temporal cortices. The temporal meta-ROI is commonly used and has shown high discriminative accuracy between AD and non-AD dementias.^4,31^ However, the temporal meta-ROI also includes medial temporal regions and therefore does not fully correspond to regions most relevant for visual read. Therefore, we additionally calculated SUVr in a temporoparietal ROI (only including regions that can contribute to a positive visual read), including the bilateral inferior, middle, and superior temporal cortices, superior parietal gyrus, inferolateral parietal lobe, and the posterior cingulate gyrus.^4^

### Statistical Analyses

Demographic characteristics between groups were compared using *t* tests, χ^2^, and Mann-Whitney *U* tests. To assess inter-reader and intra-reader reliabilities, Cohen κ coefficients were calculated. The prevalence of visual-read tau positivity was determined per diagnostic group (CU, AD, and DLB) stratified by Aβ status. Independent *t* tests were performed to compare tau-PET SUVr between visual read tau-negative and visual read tau-positive participants. To examine the correspondence of tau status defined by visual read and tau status defined by SUVr, we obtained SUVr thresholds (ROI specific) using 2 approaches: first, by fitting a Gaussian mixture model (GMM) with 2 components resulting in a threshold representing the mean of the mu of both components^32,33^ and second, by defining the threshold as mean + [2 × SD] of Aβ-negative CU participants.^4^ Percentages of concordance and discordance in tau status between visual read and the 2 SUVr thresholds were calculated. Next, we assessed associations of tau-PET visual read with age, sex, *APOE* ε4 carriership, and prospective cognitive decline in CU and AD. There was too limited power in the DLB group due to low number of tau-positive cases with DLB. Associations of tau-PET visual read (outcome) with age, sex, and *APOE* ε4 (predictors) were performed using bivariate binary logistic regressions (separate models per predictor). A multivariable logistic regression including all significant predictors was performed to test predictors' independent effects. Associations of tau-PET visual read (predictor) with prospective decline on the MMSE (outcome) were performed using age-adjusted, sex-adjusted, and education-adjusted linear mixed models (LMMs) with a random intercept (MMSE ∼ visual read × time + visual read + time + age + sex + education + (1 | participant)). A random slope (time | participant) was added if it improved model fit based on the Akaike information criterion and the χ^2^ statistic (see eMethods, links.lww.com/WNL/D110). Time reflected time between tau-PET and MMSE. Education was based on the Dutch Verhage score.^34^ Continuous variables were z transformed before model entry. To test whether SUVr was able to explain additional variance in cognitive decline within visual read tau-positive AD, an additional age-adjusted, sex-adjusted, and education-adjusted LMM with a subject-specific intercept and temporal meta-ROI SUVr, time, and an interaction term of SUVr × time was performed in tau-positive participants with AD (MMSE ∼ tau-PET SUVr × time + tau-PET SUVr + time + age + sex + education + [1 | participant]).

We used R version 4.0.3 for statistical analyses. *p* Value <0.05 was considered significant.

### Data Availability

Anonymized data that support the findings of this study are available on reasonable request from a qualified investigator.

## Results

### Participants

We included 263 participants including 147 CU participants, 97 participants with AD, and 19 participants with DLB (Table 1). By design, all participants with AD were Aβ-positive. Furthermore, 52 CU participants (36.1%) and 7 participants with DLB (36.8%) were Aβ-positive. Participants with AD were significantly younger (65.6 ± 7.6 years) compared with CU participants (70.2 ± 7.7, *p* < 0.001) and participants with DLB (69.5 ± 5.6, *p* = 0.03). There were fewer female participants in the DLB group (15.8%) compared with those in CU (49.7%, *p* = 0.01) and AD (45.4%, *p* = 0.03) groups. Moreover, there were more *APOE* ε4 carriers in the AD group (72.0%) compared with those in CU (55.9%, *p* < 0.001) and DLB (35.3%, *p* = 0.01) groups. As expected, MMSE was lower in AD (21.9 ± 4.5) and DLB (23.8 ± 4.6) groups compared with those in the CU group (28.8 ± 1.3, both *p* < 0.005). Among the 97 participants with AD, there were 9 participants with an atypical AD variant (5 posterior cortical atrophy, 2 logopenic progressive aphasia, and 2 behavioral AD).

**Table 1:**
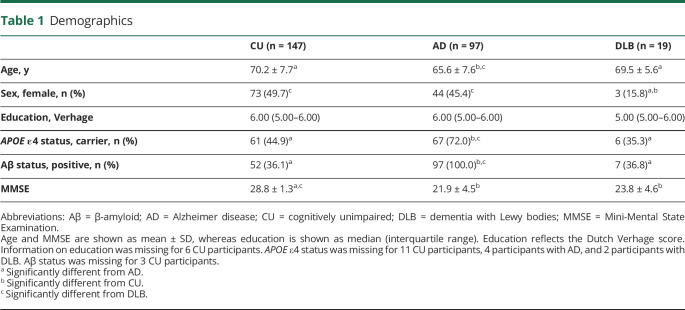
Demographics

	CU (n = 147)	AD (n = 97)	DLB (n = 19)
Age, y	70.2 ± 7.7^a^	65.6 ± 7.6^b,c^	69.5 ± 5.6^a^
Sex, female, n (%)	73 (49.7)^c^	44 (45.4)^c^	3 (15.8)^a,b^
Education, Verhage	6.00 (5.00–6.00)	6.00 (5.00–6.00)	5.00 (5.00–6.00)
*APOE* ε4 status, carrier, n (%)	61 (44.9)^a^	67 (72.0)^b,c^	6 (35.3)^a^
Aβ status, positive, n (%)	52 (36.1)^a^	97 (100.0)^b,c^	7 (36.8)^a^
MMSE	28.8 ± 1.3^a,c^	21.9 ± 4.5^b^	23.8 ± 4.6^b^

Abbreviations: Aβ = β-amyloid; AD = Alzheimer disease; CU = cognitively unimpaired; DLB = dementia with Lewy bodies; MMSE = Mini-Mental State Examination.

Age and MMSE are shown as mean ± SD, whereas education is shown as median (interquartile range). Education reflects the Dutch Verhage score. Information on education was missing for 6 CU participants. *APOE* ε4 status was missing for 11 CU participants, 4 participants with AD, and 2 participants with DLB. Aβ status was missing for 3 CU participants.

a Significantly different from AD.

b Significantly different from CU.

c Significantly different from DLB.

### Inter-reader and Intra-reader Agreements

Across all tau-PET scans (368 scans), the inter-reader agreement between the 2 nuclear medicine physicians for visual read was excellent (κ = 0.95, CI 0.91–0.99). For baseline (263 scans), 2-year follow-up (90 scans), and 4-year follow-up (15 scans) separately, comparable Cohen kappa coefficients were observed (baseline: κ = 0.95, CI 0.91–0.99; 2-year follow-up: κ = 0.96, CI 0.89–1.0; and 4-year follow-up: κ = 1.00, CI 1.0–1.0). There was disagreement between readers in only 8 scans (2.2%; 6 baseline and two 2-year follow-up scans), for which consensus reads were obtained for subsequent analyses. The 8 scans with between-reader disagreement belonged to 4 CU participants, 2 participants with AD, and 1 participant with DLB, of which 1 participant with AD had between-reader disagreement on both baseline and 2-year follow-up (consensus reads were obtained independently of each other). The final consensus read was in line with the initial read of reader 1 for 2/8 scans. Intra-reader agreement (i.e., between training set and baseline set) was excellent (κ = 0.90, CI 0.71–0.90, for both readers).

### Prevalence of Tau-PET Visual Read Positivity

**Figure 1:**
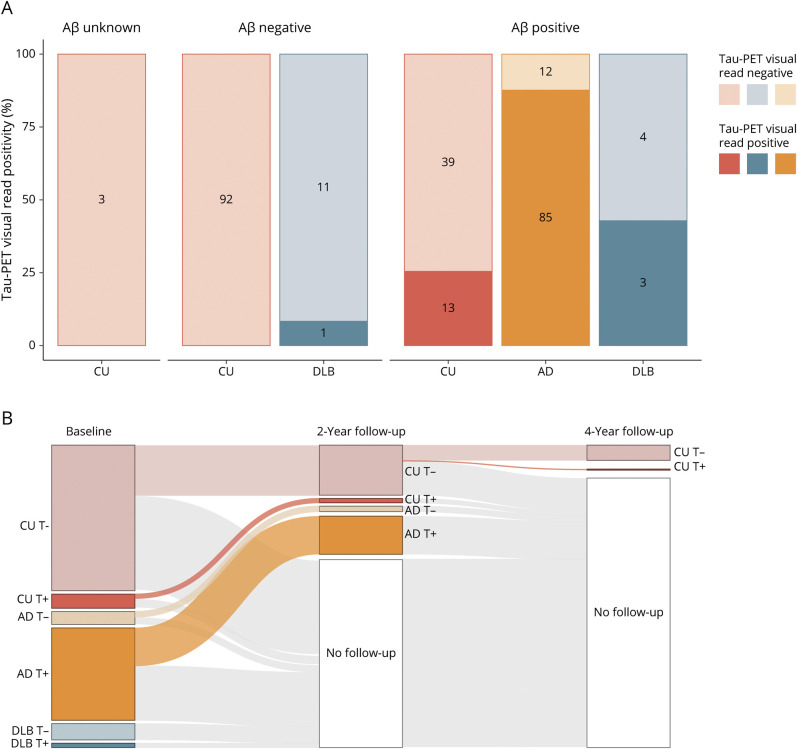
Cross-sectional and Longitudinal Tau-PET Visual Read Status (A) The prevalence of baseline tau-PET positivity stratified according to diagnostic group (CU, AD, and DLB) and Aβ status (unknown, negative, and positive) is shown. Numbers indicate the number of participants visually read as tau-negative or tau-positive within each group. (B) Tau-PET visual read status (positive [+] or negative [−]) for each diagnostic group at baseline, 2-year follow-up, and 4-year follow-up indicates that outcome of the visual read method is stable over time. The single CU participant that converted to tau-positive at 4-year follow-up was Aβ positive. Aβ = β-amyloid; AD = Alzheimer disease; CU = cognitively unimpaired; DLB = dementia with Lewy bodies.

We examined the prevalence of tau-PET visual read positivity stratified according to diagnosis (CU, AD, and DLB) and Aβ status (negative/positive) (Figure 1A). Among Aβ-negative participants, 1 participant with DLB was visually read as tau-positive (1/12 DLB [8.3%]). None of the Aβ-negative CU participants (0/92 [0%]) were visually read as tau-positive. Among Aβ-positive participants, 13 CU participants (13/52 [25.0%]), 85 participants with AD (85/97 [87.6%]), and 3 participants with DLB (3/7 [42.9%]) were visually read as tau-positive. There were 3 CU participants with unknown Aβ status, who were all tau-negative. Among the 9 participants with an atypical AD variant, all except 1 participant with logopenic progressive aphasia were tau-positive.

We next examined stability in tau-PET visual read status over time for the subset with 2-year follow-up (n = 90) and 4-year follow-up (n = 15) available. For all participants, tau-PET visual read at 2-year follow-up was identical to tau-PET visual read at baseline. At 4-year follow-up, there was 1 Aβ-positive CU participant that changed from tau-negative to tau-positive (Figure 1B).

### Comparing Tau-PET Visual Read With Tau-PET SUVr

We next compared tau-PET visual read with a semiquantitative measure of tau tracer binding (SUVr). Four scans (4/263 [1.5%], n = 3 tau-positive AD and n = 1 tau-negative DLB) did not meet scan quality criteria for SUVr due to severe motion during the scan. Reported in the text are results for temporal meta-ROI SUVr, whereas eFigure 1 (links.lww.com/WNL/D108) shows results for temporoparietal ROI SUVr.

**Figure 2:**
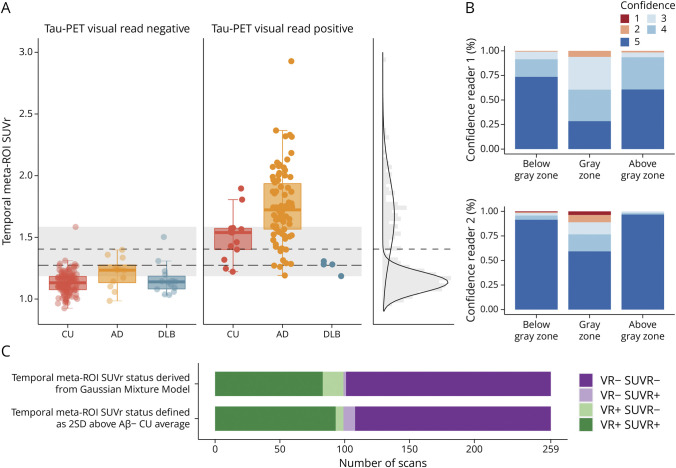
Comparing Tau-PET Visual Read With Tau-PET SUVr (A) Tau-PET SUVr in the temporal meta-ROI is plotted, stratified by diagnostic group (CU, AD, and DLB) and tau-PET visual read status (negative and positive). The short dashed line represents the SUVr cutoff derived from a GMM with the 2 Gaussian distributions plotted on the right. The long dashed line represents the SUVr cutoff defined as 2 SDs above the mean of Aβ negative CU participants. The gray zone represents visual read positive and visual read negative scans with overlapping SUVr. (B) The confidence of the 2 readers (ranging from 1 to 5) is shown for scans below the gray zone, within the gray zone, and above the gray zone. (C) The number of scans with concordant or discordant VR and SUVr status (based both GMM and mean + [2 × SD]) is shown. Aβ = β-amyloid; AD = Alzheimer disease; CU = cognitively unimpaired; DLB = dementia with Lewy bodies; GMM = Gaussian mixture model; ROI = region of interest; SUVr = standardized uptake value ratio; VR = visual read.

Compared with visual read tau-negative participants of the same diagnostic group, temporal meta-ROI SUVr was higher in visual read tau-positive CU participants (*p* < 0.001), participants with AD (*p* < 0.001), and participants with DLB (*p* = 0.03). However, there was also overlap in temporal meta-ROI SUVr between visual read tau-negative and visual read tau-positive participants, as highlighted in gray in Figure 2A. A total of 81 scans (81/259 [31.3%]) fell within this overlapping “gray zone” (SUVr 1.19–1.59). The 2 readers showed significantly lower confidence scores for scans within the gray zone compared with scans below (*p* < 0.001 for both readers) or above the gray zone (*p* < 0.001 for both readers) (Figure 2B). Of 8 scans with initial between-reader disagreement, 6 scans had SUVr values falling within the gray zone and 2 scans had SUVr values slightly below the gray zone (SUVr 1.12 and 1.17).

To define tau-PET status based on temporal meta-ROI SUVr, we identified a threshold of 1.41 SUVr derived from a GMM with 2 components (short dashed line in Figure 2A) and a threshold of 1.28 SUVr derived from the mean + [2 × SD] of Aβ-negative CU participants (long dashed line in Figure 2A). When comparing visual read tau status with SUVr tau status (taking both SUVr thresholds into account), most of the scans were concordant on tau status (234 scans concordant on all 3 tau status measures [90.4%]) (Figure 2C). Discordant visual read tau-negative SUVr tau-positive scans were observed more often when using the mean + [2 × SD] threshold (9 scans) compared with when using the GMM threshold (2 scans). To the contrary, discordant visual read tau-positive SUVr tau-negative scans were observed more often when using the GMM threshold (16 scans) compared with when using the mean + [2 × SD] threshold (6 scans). Discordance was especially noticeable in the DLB group, where visual read tau-positive participants with DLB showed generally low SUVr values.

**Figure 3:**
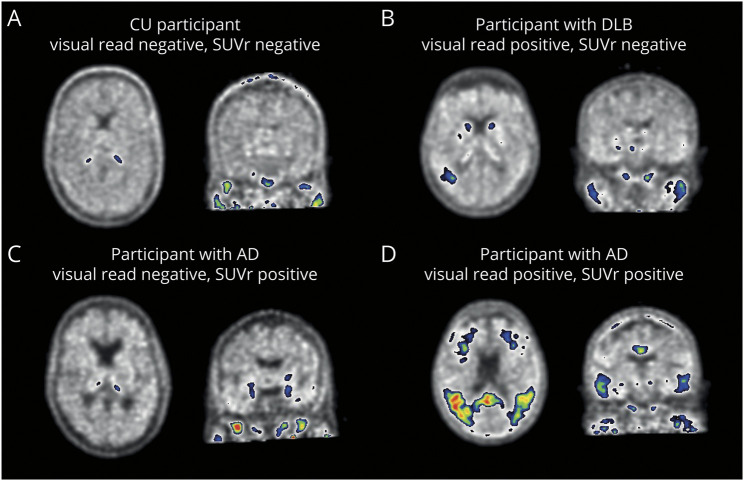
Example [^18^F]Flortaucipir PET Scans for Visual Read Shown are [^18^F]flortaucipir PET scans of 4 participants. (A) A CU participant defined as tau-negative on both visual read and SUVr. (B) A participant with DLB defined as visual read tau-positive, but SUVr negative. Increased tracer uptake was observed in only a small region, potentially resulting in low SUVr. (C) A participant with AD defined as visual read negative, but SUVr positive. Increase tracer uptake was observed isolated to the medial temporal lobe, which does not contribute to a positive tau-PET visual read. (D) A participant with AD defined as tau-positive on both visual read and SUVr. AD = Alzheimer disease; CU = cognitively unimpaired; DLB = dementia with Lewy bodies; SUVr = standardized uptake value ratio.

In Figure 3, we highlighted 4 representative scans with concordant or discordant tau status. The discordant participant with DLB (visual read tau-positive, SUVr tau-negative) showed tracer uptake in a relatively small region, potentially resulting in a low SUVr. The discordant participant with AD (visual read tau-negative, SUVr tau-positive) showed tracer uptake predominantly in the medial temporal lobe, which does not contribute to a positive visual read.

Results for temporoparietal SUVr (eFigure 1, links.lww.com/WNL/D108) were similar, but showed a slightly lower concordance between visual read and SUVr status (224 scans concordant on all 3 tau status measures [86.5%]).

### Demographic Factors Associated With Tau-PET Visual Read Status

**Figure 4:**
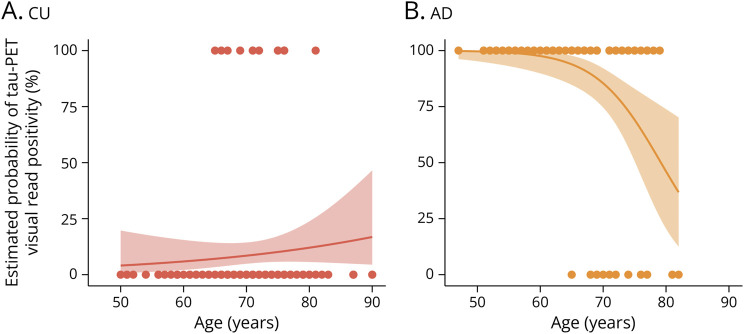
Estimated Probabilities of Tau-PET Visual Read Positivity According to Age Plotted are the predicted probabilities of tau-PET visual read positivity according to age obtained from a logistic regression between tau-PET visual read (outcome) and age (predictor). We additionally superimposed individual data points to better visualize the distribution of tau-negative and tau-positive cases according to age. A trend toward a positive association between age and probability of tau-PET positivity was observed in CU participants (A), whereas a strong negative association between age and probability of tau-PET positivity was observed in participants with AD (B). AD = Alzheimer disease; CU = cognitively unimpaired.

We next examined associations of age, sex, and *APOE* ε4 with tau-PET visual read in CU participants and those with AD (eTable 1, links.lww.com/WNL/D109). Due to the low number of tau-positive cases with DLB, these analyses could not be performed for DLB. In CU participants, *APOE* ε4 carriership was associated with a higher odds for tau positivity (odds ratio [OR] 4.15, CI 1.17–19.41, *p* = 0.04), but this effect disappeared when restricting the analysis to Aβ-positive CU participants (OR 1.56, CI 0.39–7.94, *p* = 0.55). In AD, both younger age (OR 0.82, CI 0.72–0.92, *p* = 0.001) and female sex (OR 11.26, CI 2.05–210.40, *p* = 0.02) were individually associated with a higher odds for tau positivity. When including age and sex in the same model, younger age remained associated with a higher odds for tau positivity (OR 0.84, CI 0.73–0.93, *p* = 0.004), and a trend was observed for female sex (OR 8.44, CI 1.41–162.83, *p* = 0.052). In Figure 4, we modeled the estimated probabilities of tau-PET visual read positivity according to age, showing a strong negative association between age and tau positivity in AD and a trend toward a positive association between age and tau positivity in CU.

### Association With Prospective Cognitive Decline

Finally, we tested the association of tau-PET visual read status with prospective longitudinal trajectories of cognitive decline in CU participants and those with AD. These analyses could not be performed in participants with DLB due to the low number of tau-positive cases with DLB.

**Figure 5:**
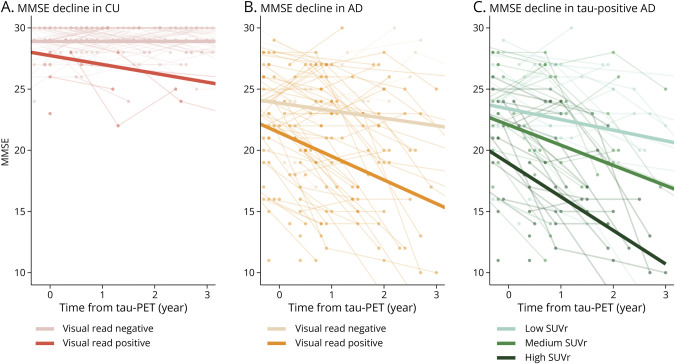
Association of Tau-PET Visual Read and Tau-PET SUVr With Prospective MMSE Spaghetti plots of longitudinal MMSE are shown. Association of tau-PET visual read status with longitudinal performance on the MMSE is shown for (A) CU participants and (B) participants with AD, with slopes from linear mixed models superimposed on the graphs. In (C) the association of temporal meta-ROI, tau-PET SUVr with longitudinal performance on the MMSE in visual read tau-positive participants with AD is shown. For visualization purposes, slopes from linear mixed models with SUVr in tertiles are superimposed on the graph. AD = Alzheimer disease; CU = cognitively unimpaired; MMSE = Mini-Mental State Examination; ROI = region of interest; SUVr = standardized uptake value ratio.

A positive tau-PET visual read was associated with worse cross-sectional MMSE in CU participants (β = −0.85, CI −1.35 to −0.35, *p* = 0.001), but no significant cross-sectional association was observed in those with AD (β = −0.48, CI −1.05 to 0.08, *p* = 0.10). Over time, a positive tau-PET visual read was associated with a steeper decline in MMSE in both CU participants (β = −0.52, CI −0.74 to −0.30, *p* < 0.001) and those with AD (β = −0.30, CI −0.58 to −0.02, *p* = 0.04) (Figure 5, A and B). For sensitivity analyses, we restricted analyses in the CU group to CU Aβ-positive participants and observed a trend-level association between a positive tau-PET visual read with worse cross-sectional MMSE (β = −0.55, CI −1.09 to −0.01, *p* = 0.06) and a significant association with steeper decline in MMSE (β = −0.40, CI −0.64 to −0.16, *p* = 0.002).

Last, we examined whether semiquantification (temporal meta-ROI SUVr) could provide prognostic information within visual read tau-positive participants with AD. Within visual read tau-positive participants with AD, higher temporal meta-ROI SUVr was associated with worse cross-sectional performance on the MMSE (β = −0.29, CI −0.50 to −0.09, *p* = 0.008) and worse prospective decline on the MMSE (β = −0.14, CI −0.20 to −0.08, *p* < 0.001) (Figure 5C).

### Classification of Evidence

This study provides Class II evidence that [^18^F]flortaucipir visual read accurately distinguishes patients with low tau-tracer binding from those with high tau-tracer binding and is associated with amyloid positivity and cognitive decline.

## Discussion

This study aimed to evaluate the performance of the US FDA–approved [^18^F]flortaucipir PET visual read method. Our results showed that the method had excellent inter-reader and intra-reader agreements, corresponded strongly with a semiquantitative approach, and was stable over time. Furthermore, a positive tau-PET visual read status was almost exclusively observed in Aβ-positive participants and was associated with prospective decline on the MMSE. Our results indicate that the visual read method is reliable and robust and that outcome of this method shows expected associations with clinically relevant variables, supporting the application of this method in clinical practice.

First, for clinical implementation, it is important that the method is reliable and accurate. A recent study validated the method to accurately detect postmortem neurofibrillary tangle pathology because positive visual reads were typically observed in postmortem Braak stage IV or higher.^13^ We add to this by showing reliability of the method with several findings. We observed a strong degree of agreement between 2 independent readers, with agreement observed in 97.8% of scans. Moreover, tau status based on visual read corresponded strongly to tau status based on a semiquantitative approach (SUVr) with concordance in tau status observed in 90.4% of scans. In addition, none of the patients with AD with available follow-up tau-PET changed in tau-PET visual read status over 2-year follow-up, indicating that outcome of the method is stable over time in clinically impaired patients. Altogether, this indicates that the visual read method accurately detects tau pathology and is reliable for clinical implementation.

Second, for clinical implementation, it is important to understand which clinically relevant factors are associated with tau-PET visual read status. Previous studies suggested that cortical Aβ is required for tau to spread beyond Braak stage IV,^35^ resulting in the expectation that a positive tau-PET visual read will be accompanied by the presence of neocortical Aβ. In line with this expectation, none of the Aβ-negative CU participants were visually read as tau-positive. However, there was 1 Aβ-negative DLB participant visually read as tau-positive. Tau positivity in Aβ-negative patients with DLB was also previously observed,^4^ and postmortem studies are needed to establish whether the tracer is truly binding to AD-type tau in these cases. As expected, tau positivity among the Aβ-positive groups was highest in AD, with 87.6% of patients with AD being visually read as tau-positive. Notably, 12.4% of patients with AD were thus tau-negative. In AD, we observed a strong decrease in prevalence of tau positivity with older age, which has also been previously reported (with comparable effect sizes) using quantitative thresholds.^36,37^ Potential explanations could be that with older age, there may be additional development of copathologies or less resilience to tau, and therefore a lower tau-threshold may be needed to result in cognitive impairment. For implementation of tau-PET visual reads in clinical practice, it will be important to further characterize these tau-negative patients with AD.

Because tau-PET is clinically expected to show strong diagnostic performance at the dementia stage of AD, it is important to note that in our study, a substantial proportion of Aβ-positive participants with DLB (42.9%) were visual read tau-positive. Postmortem studies indicated that approximately 50% of patients with DLB also have Aβ and tau pathology.^15^ Of interest, previous tau-PET studies in DLB using quantitative PET measures have generally shown minimal tracer uptake in patients with DLB.^38,39^ In this study, we also observed that SUVr of visual read tau-positive patients with DLB was low and indistinguishable from SUVr of visual read tau-negative patients with DLB. A potential explanation could be that patients with DLB have relatively focal and low amounts of tau, which is detectable by visual read, but this signal may be attenuated when assessed quantitatively within a larger region of interest. Future studies may look into potential differences in spatial patterns of tau-positive DLB and tau-positive AD to examine whether spatial information may help in the differentiation.

To compare tau-PET visual read status with tau-PET SUVr status, we used 2 threshold approaches because there is no consensus yet on the optimal threshold for defining SUVr positivity. For both approaches, a high percentage of concordance with tau-PET visual read was observed. However, differences between the SUVr approaches in the composition of concordant and discordant groups were also observed. This indicates potential difficulty when defining tau positivity based on quantification. In addition, our results showed that there was a certain amount of overlap in tau-PET SUVr (the “gray zone”) between visual read negative and visual read positive scans. Overlap in tau-PET SUVr may not be unexpected because tau-PET binding tends to have a more continuous (albeit skewed) distribution, which is in contrast to, for example, amyloid-PET, which tends to have a more bimodal distribution. Therefore, a larger “gray zone” may be expected for tau-PET than for amyloid-PET. Moreover, 6 of 8 scans with initial between-reader disagreement had SUVr values within this gray zone, and the readers' certainty was lower for scans in this gray zone. It would be of interest to examine whether providing tau-PET SUVr to the readers could result in higher confidence scores for visual assessment and thus whether SUVr could aid in the diagnostic process.

Tau-PET is expected to provide not only diagnostic but also accurate prognostic information in the clinic. Previous studies have indicated the utility of tau-PET as a prognostic marker,^9,10,14^ and we add to this by showing that tau positivity assessed by visual read is also associated with prospective cognitive decline. This is of clinical relevance, given that biomarkers that are currently used clinically (e.g., Aβ PET) show weaker associations with cognitive decline and brain atrophy, especially at the dementia stage.^9,10^ However, also within tau-positive patients with AD, large variation in cognition exists. Our results showed that within visual read tau-positive patients with AD, tau-PET SUVr was associated with prospective cognitive decline. This indicates that SUVr has potential to provide prognostic information beyond visual read, which is of interest to investigate further.

Although the visual read method is not approved for use in cognitively normal individuals, we also examined this method in a relatively large CU sample. Among Aβ-positive CU participants, 25.0% was visually read as tau-positive. This is higher compared with what has been reported using semiquantitative thresholds, which showed approximately 5%–10% tau positivity in Aβ-positive individuals.^4,5^ A potential explanation could be that our CU group partly consisted of individuals with SCD, which has been associated with increased risk of dementia.^40^ Moreover, it must be noted that the cohorts from which CU individuals were included were enriched for Aβ positivity.^41^ Over 2-year follow-up, none of the CU tau-negative participants (n = 46, of which 13 were Aβ positive) converted to tau-positive. There was 1 CU tau-negative (Aβ-positive) participant (of n = 15, of which 2 were Aβ-positive) who converted to tau-positive at 4-year follow-up. This may indicate a limited sensitivity of the visual read method to detect the earliest changes in tau pathology.^13^ Previous studies have proposed similar, though not identical, visual read schemes which (in contrast to the US FDA–approved visual read method) also include isolated medial temporal lobe binding.^42-45^ All methods seem to correspond well with quantitative measures of tracer binding. Head-to-head comparisons are needed to examine differences in sensitivity and specificity between the visual read schemes.

Strengths include the relatively large sample size, longitudinal data, and the use of both visual read and quantification. This study also has limitations. Our DLB cohort was relatively small and did not have follow-up, and we did not include other non-AD dementias, limiting the ability to test diagnostic accuracies. In addition, our cohort consisted of few atypical cases with AD. Future studies with more cases of non-AD dementia and atypical cases with AD are of interest. Furthermore, all clinically impaired participants were recruited from a tertiary memory clinic, which may limit generalizability to the general population. In addition, participants come from selected research populations, which may limit generalizability to daily practice. Future studies are encouraged to evaluate tau-PET visual reads in large, unselected cohorts, as has been done with amyloid-PET.^2^ In addition, less than 50% of patients with AD were female, which may be lower than the general population with clinical AD and should be taken into account when interpreting the data. Furthermore, we and others observed that some patients with AD are tau-negative.^4^ However, we were not able to validate whether these individuals were devoid of tau using postmortem data. Examining postmortem data of tau-negative patients with AD is important to confirm the absence of tau pathology in these cases. Finally, the inter-reader and intra-reader agreement in this study have to be cautiously interpreted because this study included highly specialized readers, and therefore the reliability metrics may not generalize to the broader community of nuclear medicine physicians. Furthermore, the intra-reader agreement may contain a learning effect.

The excellent inter-reader agreement, strong correspondence with a semiquantitative approach, and longitudinal stability indicate that the US FDA–approved visual read method is reliable and robust, supporting its clinical application. Furthermore, tau-PET visual read was associated with prospective cognitive decline, highlighting its additional prognostic potential.

## Appendix Authors

**Table:**

Name	Location	Contribution
Emma M. Coomans, MSc	Radiology & Nuclear Medicine, Vrije Universiteit Amsterdam, Amsterdam UMC location VUmc; Brain Imaging, Amsterdam Neuroscience, the Netherlands	Drafting/revision of the article for content, including medical writing for content; major role in the acquisition of data; study concept or design; and analysis or interpretation of data
Lotte A. de Koning, MSc	Radiology & Nuclear Medicine, Vrije Universiteit Amsterdam, Amsterdam UMC location VUmc; Brain Imaging, Amsterdam Neuroscience, the Netherlands	Drafting/revision of the article for content, including medical writing for content; major role in the acquisition of data; major role in the analysis or interpretation of data
Roos M. Rikken, MSc	Radiology & Nuclear Medicine, Vrije Universiteit Amsterdam, Amsterdam UMC location VUmc; Brain Imaging, Amsterdam Neuroscience, the Netherlands	Drafting/revision of the article for content, including medical writing for content; major role in the acquisition of data
Sander C.J. Verfaillie, PhD	Radiology & Nuclear Medicine, Vrije Universiteit Amsterdam, Amsterdam UMC location VUmc; Brain Imaging, Amsterdam Neuroscience; Medical Psychology, Amsterdam UMC location University of Amsterdam, the Netherlands	Study concept or design; analysis or interpretation of data
Denise Visser, MSc	Radiology & Nuclear Medicine, Vrije Universiteit Amsterdam, Amsterdam UMC location VUmc; Brain Imaging, Amsterdam Neuroscience, the Netherlands	Drafting/revision of the article for content, including medical writing for content; major role in the acquisition of data
Anouk den Braber, PhD	Alzheimer Center Amsterdam, Neurology, Vrije Universiteit Amsterdam, Amsterdam UMC location VUmc; Neurodegeneration, Amsterdam Neuroscience; Department of Biological Psychology, Vrije Universiteit Amsterdam, the Netherlands	Drafting/revision of the article for content, including medical writing for content; major role in the acquisition of data
Jori Tomassen, MSc	Alzheimer Center Amsterdam, Neurology, Vrije Universiteit Amsterdam, Amsterdam UMC location VUmc; Neurodegeneration, Amsterdam Neuroscience, the Netherlands	Drafting/revision of the article for content, including medical writing for content; major role in the acquisition of data
Marleen van de Beek, PhD	Alzheimer Center Amsterdam, Neurology, Vrije Universiteit Amsterdam, Amsterdam UMC location VUmc; Neurodegeneration, Amsterdam Neuroscience, the Netherlands	Drafting/revision of the article for content, including medical writing for content; major role in the acquisition of data
Lyduine E. Collij, PhD	Radiology & Nuclear Medicine, Vrije Universiteit Amsterdam, Amsterdam UMC location VUmc; Brain Imaging, Amsterdam Neuroscience, the Netherlands	Study concept or design; analysis or interpretation of data
Afina W. Lemstra, MD, PhD	Alzheimer Center Amsterdam, Neurology, Vrije Universiteit Amsterdam, Amsterdam UMC location VUmc; Neurodegeneration, Amsterdam Neuroscience, the Netherlands	Drafting/revision of the article for content, including medical writing for content; major role in the acquisition of data
Albert D. Windhorst, PhD	Radiology & Nuclear Medicine, Vrije Universiteit Amsterdam, Amsterdam UMC location VUmc; Brain Imaging, Amsterdam Neuroscience, the Netherlands	Drafting/revision of the article for content, including medical writing for content; major role in the acquisition of data
Frederik Barkhof, MD, PhD	Radiology & Nuclear Medicine, Vrije Universiteit Amsterdam, Amsterdam UMC location VUmc; Brain Imaging, Amsterdam Neuroscience, the Netherlands; Queen Square Institute of Neurology and Centre for Medical Image Computing, University College London, United Kingdom	Drafting/revision of the article for content, including medical writing for content
Sandeep S.V. Golla, PhD	Radiology & Nuclear Medicine, Vrije Universiteit Amsterdam, Amsterdam UMC location VUmc; Brain Imaging, Amsterdam Neuroscience, the Netherlands	Drafting/revision of the article for content, including medical writing for content
Pieter Jelle Visser, MD, PhD	Alzheimer Center Amsterdam, Neurology, Vrije Universiteit Amsterdam, Amsterdam UMC location VUmc; Neurodegeneration, Amsterdam Neuroscience; Alzheimer Center Limburg, School for Mental Health and Neuroscience, Maastricht University, the Netherlands; Division of Neurogeriatrics, Department of Neurobiology, Care Sciences and Society, Karolinska Institutet, Stockholm, Sweden	Drafting/revision of the article for content, including medical writing for content
Philip Scheltens, MD, PhD	Alzheimer Center Amsterdam, Neurology, Vrije Universiteit Amsterdam, Amsterdam UMC location VUmc; Neurodegeneration, Amsterdam Neuroscience, the Netherlands	Drafting/revision of the article for content, including medical writing for content
Wiesje M. van der Flier, PhD	Alzheimer Center Amsterdam, Neurology, Vrije Universiteit Amsterdam, Amsterdam UMC location VUmc; Neurodegeneration, Amsterdam Neuroscience; Department of Epidemiology & Data Science, Vrije Universiteit Amsterdam, Amsterdam UMC, the Netherlands	Drafting/revision of the article for content, including medical writing for content
Rik Ossenkoppele, PhD	Alzheimer Center Amsterdam, Neurology, Vrije Universiteit Amsterdam, Amsterdam UMC location VUmc; Neurodegeneration, Amsterdam Neuroscience, the Netherlands; Clinical Memory Research Unit, Lund University, Sweden	Drafting/revision of the article for content, including medical writing for content
Bart N.M. van Berckel, MD, PhD	Radiology & Nuclear Medicine, Vrije Universiteit Amsterdam, Amsterdam UMC location VUmc; Brain Imaging, Amsterdam Neuroscience, the Netherlands	Drafting/revision of the article for content, including medical writing for content; major role in the acquisition of data; study concept or design; and analysis or interpretation of data
Elsmarieke van de Giessen, MD, PhD	Radiology & Nuclear Medicine, Vrije Universiteit Amsterdam, Amsterdam UMC location VUmc; Brain Imaging, Amsterdam Neuroscience, the Netherlands	Drafting/revision of the article for content, including medical writing for content; major role in the acquisition of data; study concept or design; and analysis or interpretation of data

## Glossary

Aβ: β-amyloid
AD: Alzheimer disease
CU: cognitively unimpaired
DLB: dementia with Lewy bodies
US FDA: US Food and Drug Administration
GMM: Gaussian mixture model
LMM: linear mixed model
MCI: mild cognitive impairment
MMSE: Mini-Mental State Examination
OR: odds ratio
ROI: region of interest
SCD: subjective cognitive decline
SUVr: standardized uptake value ratio
